# Colour opponency is widespread across the mouse subcortical visual system and differentially targets GABAergic and non-GABAergic neurons

**DOI:** 10.1038/s41598-023-35885-z

**Published:** 2023-06-08

**Authors:** R. C. Feord, A. Gomoliszewska, A. Pienaar, J. W. Mouland, T. M. Brown

**Affiliations:** Centre for Biological Timing, Faculty of Biology, Medicine and Health, University of Manchester, Manchester, UK

**Keywords:** Neuroscience, Visual system, Colour vision, Thalamus

## Abstract

Colour vision plays many important roles in animal behaviour but the brain pathways processing colour remain surprisingly poorly understood, including in the most commonly used laboratory mammal, mice. Indeed, particular features of mouse retinal organisation present challenges in defining the mechanisms underlying colour vision in mice and have led to suggestions that this may substantially rely on ‘non-classical’ rod-cone opponency. By contrast, studies using mice with altered cone spectral sensitivity, to facilitate application of photoreceptor-selective stimuli, have revealed widespread cone-opponency across the subcortical visual system. To determine the extent to which such findings are truly reflective of wildtype mouse colour vision, and facilitate neural circuit mapping of colour-processing pathways using intersectional genetic approaches, we here establish and validate stimuli for selectively manipulating excitation of the native mouse S- and M-cone opsin classes. We then use these to confirm the widespread appearance of cone-opponency (> 25% of neurons) across the mouse visual thalamus and pretectum. We further extend these approaches to map the occurrence of colour-opponency across optogenetically identified GABAergic (GAD2-expressing) cells in key non-image forming visual centres (pretectum and intergeniculate leaflet/ventral lateral geniculate; IGL/vLGN). Strikingly, throughout, we find S-ON/M-OFF opponency is specifically enriched in non-GABAergic cells, with identified GABAergic cells in the IGL/VLGN entirely lacking this property. Collectively, therefore, we establish an important new approach for studying cone function in mice, confirming a surprisingly extensive appearance of cone-opponent processing in the mouse visual system and providing new insight into functional specialisation of the pathways processing such signals.

## Introduction

Despite the central role of colour discrimination in vision and visually guided behaviours^1–4^, current understanding of colour processing in the brain and the underlying neural pathways is still surprisingly limited. Indeed, as discussed below, a notable barrier to addressing these deficits derives from the fact that our most tractable and widely used mammalian laboratory model organism, the mouse, presents particular challenges in the study of colour vision.

The starting point for any colour-based mechanism is a comparison between the activation states of two or more spectrally distinct photopigments. In mammals, colour vision is therefore classically considered to originate via opponent processing of signals provided by different cone opsin classes^3,4^. Mice, like most other mammals, possess two such classes—the short (S-) and medium (M-) wavelength sensitive cone opsins which are, respectively, maximally sensitive in UV and green portions of the spectrum (λ_Max_ = 365 nm and 511 nm)^5–7^. Unusually, however, many mouse cones co-express both cone opsins, albeit with a dorsal–ventral retinal gradient such that the dorsal retina contains mainly M-cones with a small population of ‘pure’ S-cones, while the ventral retina is dominated by cones that express S-opsin and little or no M-opsin^8–11^. Traditionally, such an arrangement was considered to significantly limit the capacity for mouse colour vision, although substantial evidence has now emerged for the presence of UV-green wavelength discrimination at the behavioural level^12^ and for neurons in the retina and brain that exhibit opposing responses to such short and longer wavelength light^13–20^. The mechanisms proposed to account for such responses have varied quite considerably, however, not least because of the widespread appearance of colour opponency in the ventral retina (where M-opsin expression is scarce) and behavioural colour discrimination in the corresponding upper visual field. Accordingly, studies have suggested that rods, whose spectral sensitivity strongly overlaps with M-opsin, may play important roles in mouse colour vision by acting in opposition to S-cone signals^17,19^.

Directly resolving the photoreceptive mechanisms regulating colour vision in mice (and reliably assessing the occurrence of colour opponency at the level of retina or brain targets) has proven challenging. To date most studies have simply compared responses to UV or ‘green’ light, in some cases on a background of the alternate wavelength^12–19^. Given the strong overlap of rod and M-opsin spectral sensitivities, distinguishing between these two photoreceptor classes as the origin of any green responses relies on the assumption that rod responses become saturated under high enough background light levels. However, recent data in mice indicate that rods can in fact continue to respond even under very high background light levels^21^. A further challenge for such approaches is that all opsins retain modest UV sensitivity due to their β-absorption band^22,23^ such that stimuli targeted to the S-opsin will also likely provide sufficient contrast to activate rod and/or M-opsin. Finally it is important to consider that UV and ‘green’ stimuli are likely to differentially engage melanopsin phototransduction, which could further complicate interpretation of any response (especially for slower/longer duration stimuli^20,24–27^).

To avoid the issues noted above, in our previous studies of colour-discrimination mechanism in mice, we have used animals in which the native M-opsin is replaced by the human L-cone opsin (*Opn1mw*^*R*^^28^). By employing multispectral (3 or more primary) stimuli, the resulting shift in cone spectral sensitivity in such animals makes it possible to selectively modulate excitation of individual photoreceptor classes using the principles of ‘silent substitution’^29–32^. These experiments have revealed a remarkably widespread appearance of cone-dependent colour opponency across subcortical targets including the visual thalamus and regions involved in non-image forming responses such as the suprachiasmatic nucleus and pretectal olivary nucleus (PON).

While existing data has not yet revealed any overt alterations in cone opsin expression or function in *Opn1mw*^*R*^ mice^28,30,33^, a key question arising from the studies highlighted above, is whether the features and roles of cone-dependent colour vision revealed in *Opn1mw*^*R*^ mice are truly reflective of those in their wildtype counterparts. Moreover, many of the retinorecipient target regions where we observe significant populations of cone opponent neurons are highly heterogeneous in terms of neurochemical phenotype and downstream projection target^34–36^. It remains unclear whether the relevant cell types and pathways differentially process colour signals to support different behavioural or physiological functions. The widespread availability of intersectional genetics tools in mice (e.g. cell-type specific drivers and conditional reporter constructs for cell identification and manipulation^37^) makes that latter question tractable, however, employing these in *Opn1mw*^*R*^ mice imposes additional burdens in terms of generating the relevant experimental animals.

Here then we set out to address these issues, by first establishing approaches to reliably isolate and study cone-based responses in mice with native cone opsin expression. We then demonstrate their utility for providing new insights into visual circuit function by employing these approaches alongside optogenetic identification of GABAergic neurons in the mouse brain.

## Results

### Cone inputs to neurons in mouse pretectum and visual thalamus

We first set out to evaluate the influence of cone photoreceptive signals and, in particular, the prevalence of cone opponent processing across key subcortical visual targets in the mouse brain. To this end, we adapted approaches we have previously used to successfully isolate cone-based responses in animals with altered cone spectral sensitivity (*Opn1mw*^*R*^ ^29–32,38^) for use in wildtype mice. Starting with a polychromatic background stimuli that provided a relative pattern of photoreceptor activation equivalent to a mouse’s experience of natural daylight, we designed a set of stimulus pairs that preferentially differed in brightness for M- and/or S-cone opsin (± 63% contrast relative to background; Fig. S1). Transitions between these stimulus pairs, presented as a squarewave modulation, therefore provided cycles of positive and negative contrast steps for one or both cone opsins (in unison or antiphase) while maintaining a consistent mean irradiance across each stimulus cycle for all stimulus pairs. While it is readily achievable to independently modulate brightness for the two cone opsin classes in this manner (and to effectively silence any responses arising from melanopsin), the close spectral sensitivity of rhodopsin means stimuli targeting the M-cone opsin are also associated with modest rod contrasts (28–35% Michelson). Accordingly, to limit any off target effects that might be ascribed to rods, we started by testing responses to transitions between these stimulus pairs under high background light levels (> 10^14^ rod-effective photons/cm^2^/s) that would be expected to strongly suppress rod responses^21,29,39^.

**Figure 1:**
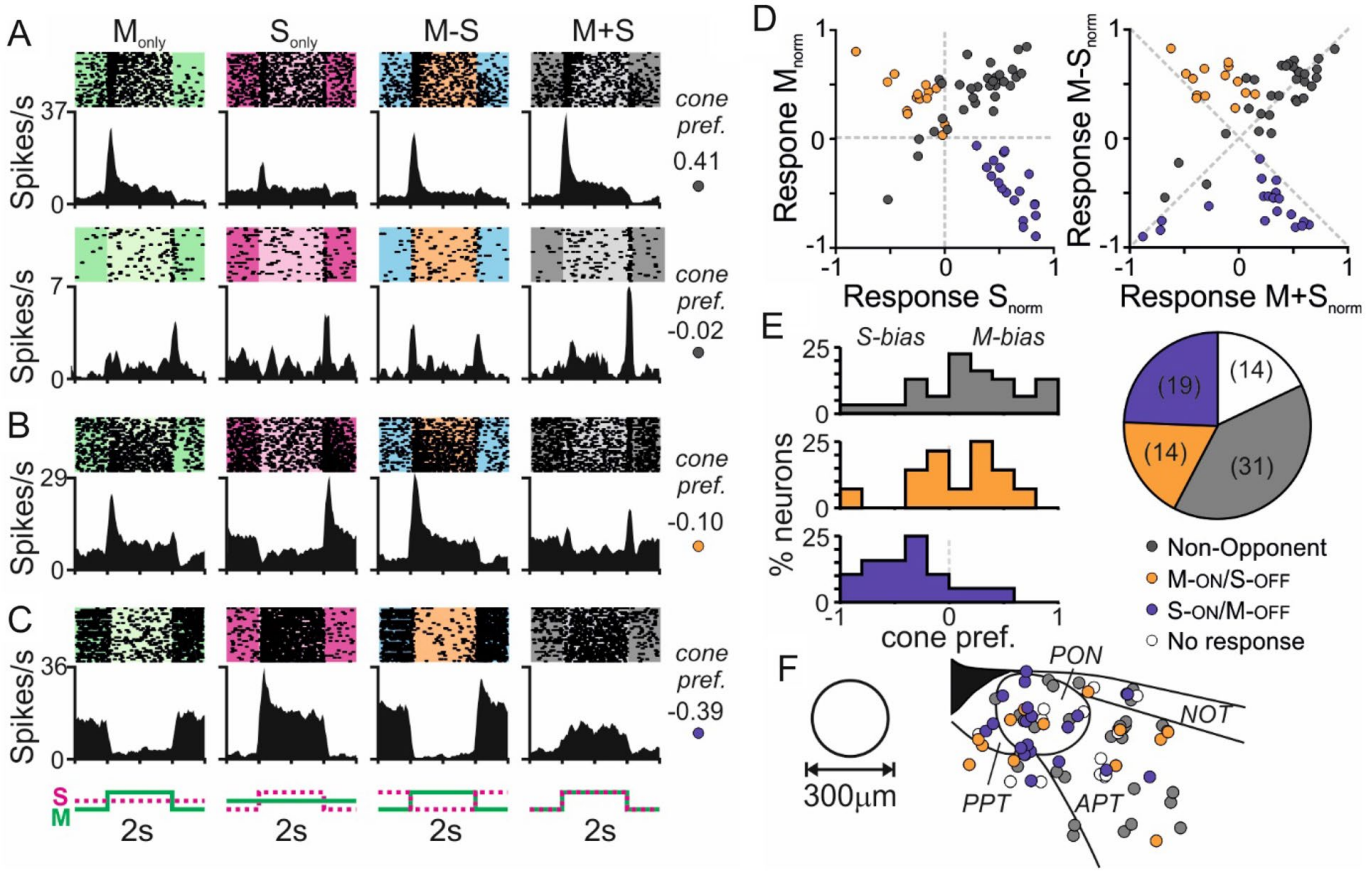
Colour opponency in subsets of neurons across the mouse pretectum. (**A**–**C**) Responses to 63% contrast cone-modulating stimuli (spike rasters and corresponding histograms) from representative mouse pretectal neurons (each cell on a different horizontal row) classified as non-opponent (**A**), opponent M-ON/S-OFF (**B**) or S-ON/M-OFF (**C**). Insets to the right of each set of traces provided the calculated cone preference for each cell ([M_R_ − S_R_]/[M_R_ + S_R_]), where a value of 0 indicates equal response to either cone type and values of − 1 and + 1 respectively indicate pure S- or M-opsin driven response. (**D**) Normalised firing responses across all responsive pretectal neurons (n = 64 from 14 recordings) for S_Only_ vs. M_Only_ (left panel) and M + S vs. M − S stimuli (right panel) subdivided according to cell classification. (**E**) Distribution of cone opsin preference (defined as above) for non-opponent (top, n = 31), M-ON/S-OFF (middle, n = 14) and S-ON/M-OFF (bottom, n = 19) pretectal neurons. Insight pie-chart illustrates proportions of cells in each class as a function of all visually responsive pretectal neurons recorded. (**F**) Projected anatomical locations of all neurons contributing to (**A**–**D**). *APT* anterior pretectum, *NOT* nucleus of the optic tract, *PON* pretectal olivary nucleus, *PPT* posterior pretectum.

We initially evaluated responses to these cone-directed stimuli (full field, 0.25 Hz squarewave modulation) across neurons in the mouse PON and surrounding pretectal region, detected during 32-channel multielectrode recordings. From fourteen such recordings, we isolated a total of 78 light responsive neurons (based on the presence of reproducible responses to bright light steps applied from a background of darkness). The majority of such cells (n = 64/78) also exhibited reliable responses to the cone-directed stimuli described above, indicating robust input from M- and/or S-cones. Consistent with data from pretectal recordings in *Opn1mw*^*R*^ mice^29^, among the responding cells we identified a variety of response types. The most common type of response (n = 31; ‘non-opponent’) was characterised by qualitatively similar changes in firing rate (overwhelmingly ‘ON’-type responses; n = 27/31) to contrast steps preferentially targeting just M- or S-cone opsin (M_Only_ and S_Only_ respectively; Fig. 1A&D). Importantly, however, we also found many cells exhibiting clear evidence of colour opponency with either ‘ON’ responses to M_Only_ and OFF responses S_Only_ stimuli (Fig. 1B; M-ON/S-OFF, n = 14) or the converse S-ON/M-OFF type response (Fig. 1C; n = 19). Consistent with these properties, such colour opponent pretectal neurons reliably exhibited larger modulations in firing rate when presented with contrast steps that modulated M- and S-cone opsin in antiphase (M − S; producing large changes in colour without changing overall luminance) and much weaker modulations in firing for achromatic (M + S) luminance contrast (Fig. 1B–D; Fig. S2A).

Across the cell populations described above, we found some variability in the relative amplitude of M- vs. S-cone opsin driven responses (Fig. 1D,E), although these were, on average, relatively evenly matched for non-opponent and M-ON/S-OFF cells and often somewhat S-opsin biased in the case of S-ON/M-OFF cells. Importantly, in addition to confirming a strong enrichment of colour opponent (especially S-ON/M-OFF) neurons in the PON region (Fig. 1F), the prevalence and properties of colour opponent neurons detected here were equivalent to those observed previously in the pretectum of *Opn1mw*^*R*^ mice^29^, when directly compared against responses to functionally near-identical cone modulating stimuli (Fig. S2A–C). We did, however, note that among non-opponent neurons, responses to M_Only_ stimuli tended to be stronger than for equivalent stimuli applied to *Opn1mw*^*R*^ mice (Fig. S2D), such that across the population there were fewer strongly S-cone biased cells then observed in those human cone knockin animals (Fig. S2E).

**Figure 2:**
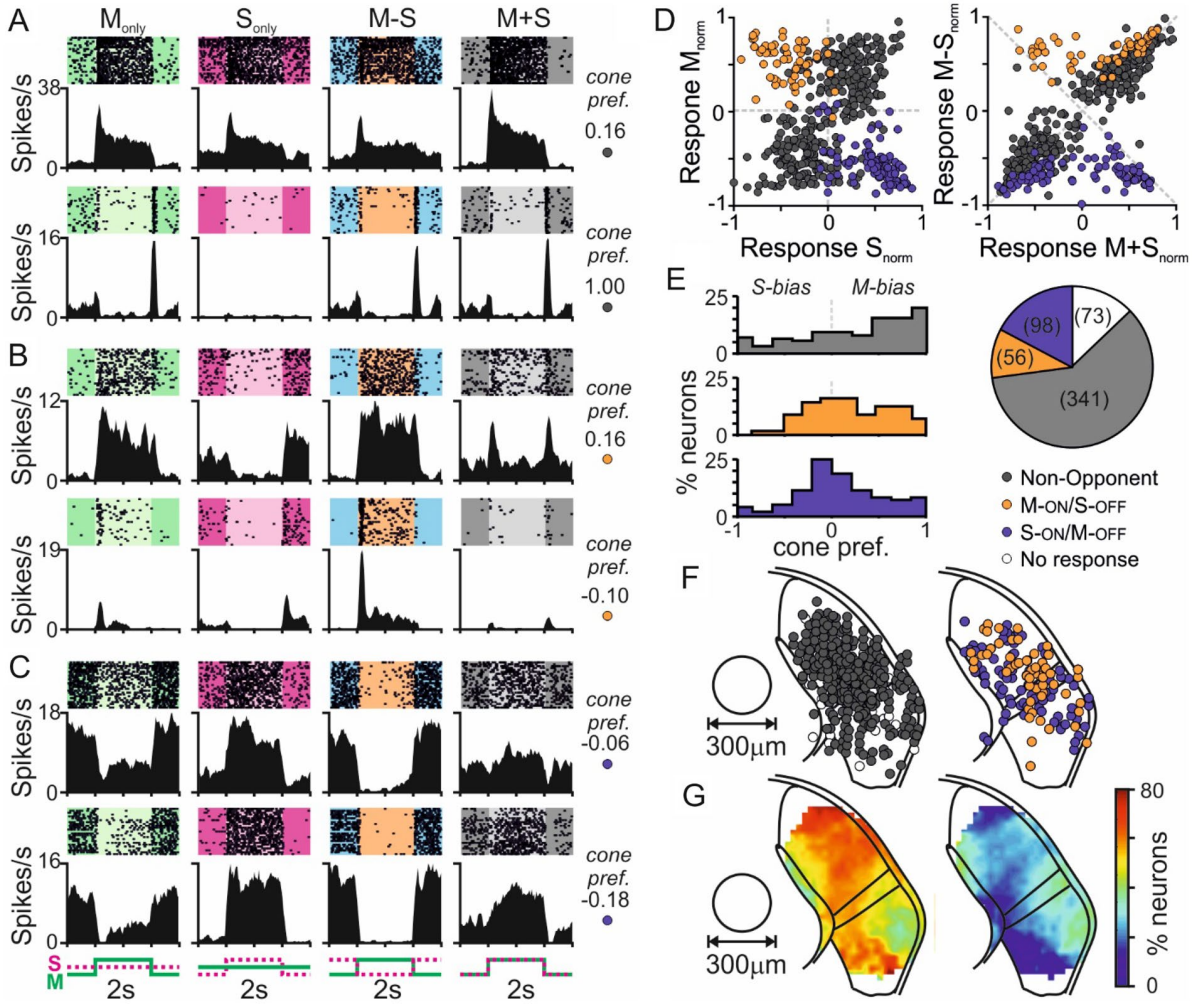
Colour opponency in subsets of neurons across the mouse LGN. (**A**–**C**) Responses to 63% contrast cone-modulating stimuli (spike rasters and corresponding histograms) from two representative mouse LGN neurons (each cell on a different horizontal row) classified as non-opponent (**A**), opponent M-ON/S-OFF (**B**) or S-ON/M-OFF (**C**). Insets to the right of each set of traces provided the calculated cone preference for each cell ([M_R_ − S_R_]/[M_R_ + S_R_]). (**D**) Normalised firing responses across all responsive LGN neurons (n = 495 from 50 recordings) for S_Only_ vs. M_Only_ (left panel) and M + S vs. M − S stimuli (right panel) subdivided according to cell classification. (**E**) Distribution of cone opsin preference (defined as above) for non-opponent (top, n = 341), M-ON/S-OFF (middle, n = 56) and S-ON/M-OFF (bottom, n = 98) LGN neurons. Insight pie-chart illustrates proportions of cells in each class as a function of all visually responsive LGN neurons recorded. (**F**) Projected anatomical locations of all neurons contributing to (**A**–**D**) (left non-opponent and non-responsive cells, right opponent neurons), mapped onto a standardised LGN template. (**G**) Prevalence of non-opponent (left) vs. opponent (right) neurons as a function of anatomical location in the LGN (binned with a 150 µm radius moving window); proportion expressed relative to total number of neurons (i.e. all cells from (**F**), including LGN cells that did not responses to cone-modulating stimuli).

We next went on to sample neurons more extensively from the lateral geniculate nuclei (LGN) complex (including dorsal part, dLGN and intergeniculate leaflet/ventral portion, IGL/vLGN). From fifty multielectrode recordings targeting the LGN we isolated n = 568 light responsive cells, of which a high proportion (n = 495; ~ 87%) responded to our cone-directed stimuli. As above, responsive neurons exhibited a variety of response type including cells exhibiting non-opponent cone-driven responses (Fig. 2A) as well as cells displaying M-ON/S-OFF (Fig. 2B) or S-ON/M-OFF colour opponency (Fig. 2C). In line with data obtained from the pretectum, non-opponent ON or OFF responses were most common (n = 183 & n = 156 respectively) but there was also a substantial proportion of cells with M-ON/S-OFF or S-ON/M-OFF opponency (n = 56 & 98 respectively; collectively ~ 31% of responsive neurons; Fig. 2D,E). Across this population of colour opponent cells, the relative magnitudes of S- and M-cone opsin driven responses were, on average, evenly matched (Fig. 2E). Moreover, as above, the prevalence and properties of such LGN opponent neurons closely resembled those observed previously in the LGN of *Opn1mw*^*R*^ mice^30^, when directly compared against responses to functionally similar cone modulating stimuli (aside from small differences in response to M/L + S luminance modulations; Fig. S3A–C). Also consistent with findings in *Opn1mw*^*R*^ mice, we found that colour opponent neurons were strongly enriched in the region of the IGL and medial portions of the dLGN of wildtype mice (Fig. 2F,G). Here, however, we also found a strong enrichment of colour-opponent neurons in the lateral segment of the vLGN (a region that was not extensively sampled in our previous work in *Opn1mw*^*R*^ mice^30^).

Collectively then, the data presented here indicate that previous reports of widespread cone-driven colour opponency across the mouse early visual system^29,30,32^ provide a true reflection of the extent of chromatic processing in mouse subcortical regions. However, as noted above for the pretectum, comparison of data for LGN non-opponent neurons against their counterparts in *Opn1mw*^*R*^ mice recorded under similar conditions, revealed a tendency for a greater bias towards M-cone/reduced bias towards S-cone opsin directed stimuli in wildtype mice (Fig. S3D,E). Given that the M-cone opsin directed stimuli employed here provided modest rod contrast (whereas L-cone directed stimuli used in *Opn1mw*^*R*^ mice did not), these data raise the possibility that, despite the high background light levels used, rods may contribute to some of the observed responses.

### Contribution of rods to photopic subcortical visual responses

To provide insight into the possibility that incomplete saturation/adaption might allow rods to contribute to responses evoked by our M-cone-directed stimuli, we next evaluated neural responses to such stimuli in mice lacking functional cone photoreception (*Cnga3*^*−/−*^^40^). In 32 channel multielectrode recordings for the LGN of nine *Cnga3*^*−/−*^ mice, we isolated n = 44 neurons that exhibited reproducible responses to light steps applied from a background of darkness. We then compared responses of such cells to the same cone-isolating stimuli used above with those evoked by spectrally neutral (‘All opsin’) modulations providing 20–96% contrast. Given the lack of cone function in these animals and temporal properties of the stimulus (faster than those which melanopsin can reliably respond to^26,29,41^), this latter stimulus should reveal the contrast response relationship for any rod-driven responses (should they be apparent under our experimental conditions).

**Figure 3:**
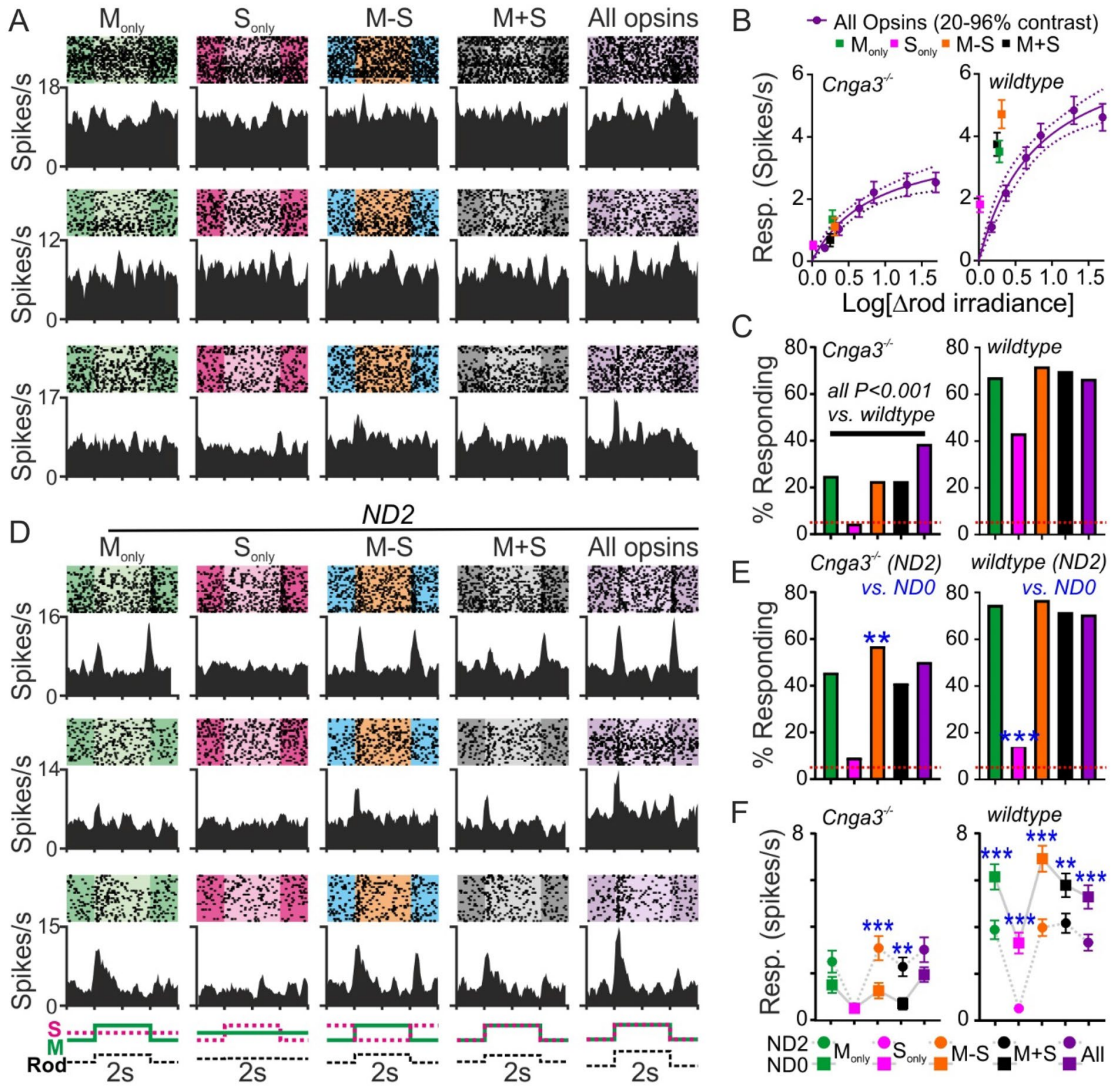
Rod influences on mouse LGN neuron responses under photopic conditions. (**A**, **D**) Responses to 63% contrast cone-directed and spectrally neutral (‘All opsin’) stimuli from three representative *Cnga3*^*−/−*^ mouse LGN neurons at full intensity (**A**; ND0) or 100-fold reduced intensity (**D**; ND2). Spike rasters and corresponding histograms for each cell plotted on a different horizontal row. (**B**) Mean ± SEM responses to cone-directed and All opsin contrast (20–96%) plotted as a function of log change in rod-effective irradiance for light responsive LGN neurons in *Cnga3*^*−/−*^ (left; n = 44 cells from 9 recordings) and wildtype (right; n = 150 cells from 13 recordings) mice. Data for spectrally neutral stimuli fit with a 2-parameter saturating function, with extra-sum-of-squares F-test to test deviation from this relationship for cone-directed stimuli (*Cnga3*^*−/−*^ : F_2,436_ = 1.15, P = 0.39; wildtype: F_2,1496_ = 31.41, P < 0.001). (**C**, **E**) Proportions of neurons exhibiting significant (P > 0.05) response to 63% contrast cone-modulating and All opsin stimuli in *Cnga3*^*−/−*^ (left) and wildtype (right) mouse LGN at ND0 (**C**) and ND2 (**E**). Data derived from same populations as (**B**) except wildtype ND2 (n = 96 light responsive neurons from 9 recordings), in (**C**) percent responding compared across genotypes, in E percent responding at ND2 compared against ND0 (both Fisher’s exacta test). (**F**) Mean ± SEM responses to 63% cone-directed and all opsin contrast for LGN neurons responding at ND0 and/or ND2 in *Cnga3*^*−/−*^ (left, n = 37) and wildtype (right; n = 93) mice. Data analysed by 2-way RM ANOVA (*Cnga3*^*−/−*^—ND: F_1, 180_ = 34.4, P < 0.001, Stim: F_4, 180_ = 6.5, P < 0.001, Interaction: F_4, 180_ = 2.7, P = 0.03; Wildtype—ND: F_1, 460_ = 124.8, P < 0.001, Stim: F_4, 460_ = 14.6, P < 0.001, Interaction: F_4, 460_ = 1.5, P = 0.22) with Sidak’s post-test. Throughout *P < 0.05, **P < 0.01 and ***P < 0.001.

As expected for the high photopic light levels used here, the majority of LGN cells in *Cnga3*^*−/−*^ mice lacked any response to our cone-directed stimuli, although we occasionally saw weak responses to the M-cone directed stimuli and (more commonly) to the higher contrast All-opsin stimuli (Fig. 3A). Indeed, across the population of light responsive cells, the mean peak-trough firing rate modulation associated with the presentation of these All-opsin stimuli increased in a predictable manner as a function of increasing contrast (Fig. 3B). Moreover, when we expressed the contrast provided by the cone-directed stimuli in terms of the associated effective change in irradiance for rods, the corresponding response magnitudes were statistically indistinguishable from those predicted by this All-opsin contrast-response function (Fig. 3B; F-test, P = 0.39).

In sum, the data described above provide good confidence that our stimulus calculations and calibration are reliable. More importantly, however, they also support previous suggestions ^21^ that rods can remain partially responsive even under very high light levels. Accordingly, while the proportion of *Cnga3*^*−/−*^ LGN cells exhibiting statistically significant modulations in firing rate (χ^2^-periodogram, P < 0.05) was at the level of chance for the S_Only_ stimulus (n = 2/44; < 5%), around one quarter of recorded neurons exhibited significant, albeit weak, modulations in firing rate for our (higher rod contrast) M-cone opsin directed stimuli (Fig. 3C). Unsurprisingly, these proportions of responding cells were all significantly lower than among cells recorded from the LGN in wildtype mice using the same protocol (n = 150 cells from 13 recordings), where the mean response amplitudes to cone directed stimuli were both far larger than in *Cnga3*^*−/−*^ LGN cells and than that predicted from the wildtype populations response to All opsin contrast (Fig. 3B,C). Qualitatively similar results were obtained when comparing responses to cone-directed stimuli in the pretectum of wildtype and *Cnga3*^*−/−*^ mice (n = 112 cells from 7 recordings; Fig. S4).

Collectively then, while the analysis above clearly confirms that the responses evoked by cone-directed stimuli in wildtype mice are indeed dominated by cone-derived signals, our results from *Cnga3*^*−/−*^ recordings indicate that rod contributions to M-cone opsin directed stimuli cannot be entirely excluded. To provide further insight into the extent to which any such influence might impact interpretation of experiments seeking to isolate cone-based responses in mice, we first evaluated the extent to which such effects are sensitive to background irradiance. To this end, we also tested the same cone-directed stimuli and the equivalent all opsin contrast stimulus at a 100-fold reduced irradiance—the lower end of the photopic range but still above the nominal saturation point for rods (ND2; > 10^12^ rod-effective photons/cm^2^/s).

As expected, based on previous data^21^, we found clear evidence of increased rod intrusion in our recordings from the LGN of *Cnga3*^*−/−*^mice when stimuli were delivered at ND2 (Fig. 3D). Hence, the proportion of *Cnga3*^*−/−*^ LGN neurons showing significant modulations in firing rate following M-cone directed stimuli increased compared to that at ND0 (especially for the M − S stimulus that was associated with the highest rod contrast; Fig. 3E). Further, among *Cnga3*^*−/−*^ LGN neurons that exhibited significant responses to one or more test stimuli, the amplitudes of the resulting firing rate modulations for M-cone directed stimuli were increased at ND2 (Fig. 3F). By contrast, for a subset of LGN cells in wildtype mice that were tested at both background light intensities (n = 96 light responsive neurons from 9 recordings; Fig. S5), the proportion of cells with significant responses fell for the S_Only_ stimulus, but not other stimuli (Fig. 3E). Moreover, among responding LGN cells in wildtype mice, response amplitudes were significantly reduced for all test stimuli, compared to the same cell’s responses at ND0 (Fig. 3F). Collectively, these latter data are indicative of a reduction in the magnitude of cone-based responses at this reduced background light intensity. Since our recordings in *Cnga3*^*−/−*^ indicate an increase in rod-based responses at ND2, these findings imply that responses to M-cone directed stimuli may become increasingly contaminated by rod signals at lower photopic background irradiances.

**Figure 4:**
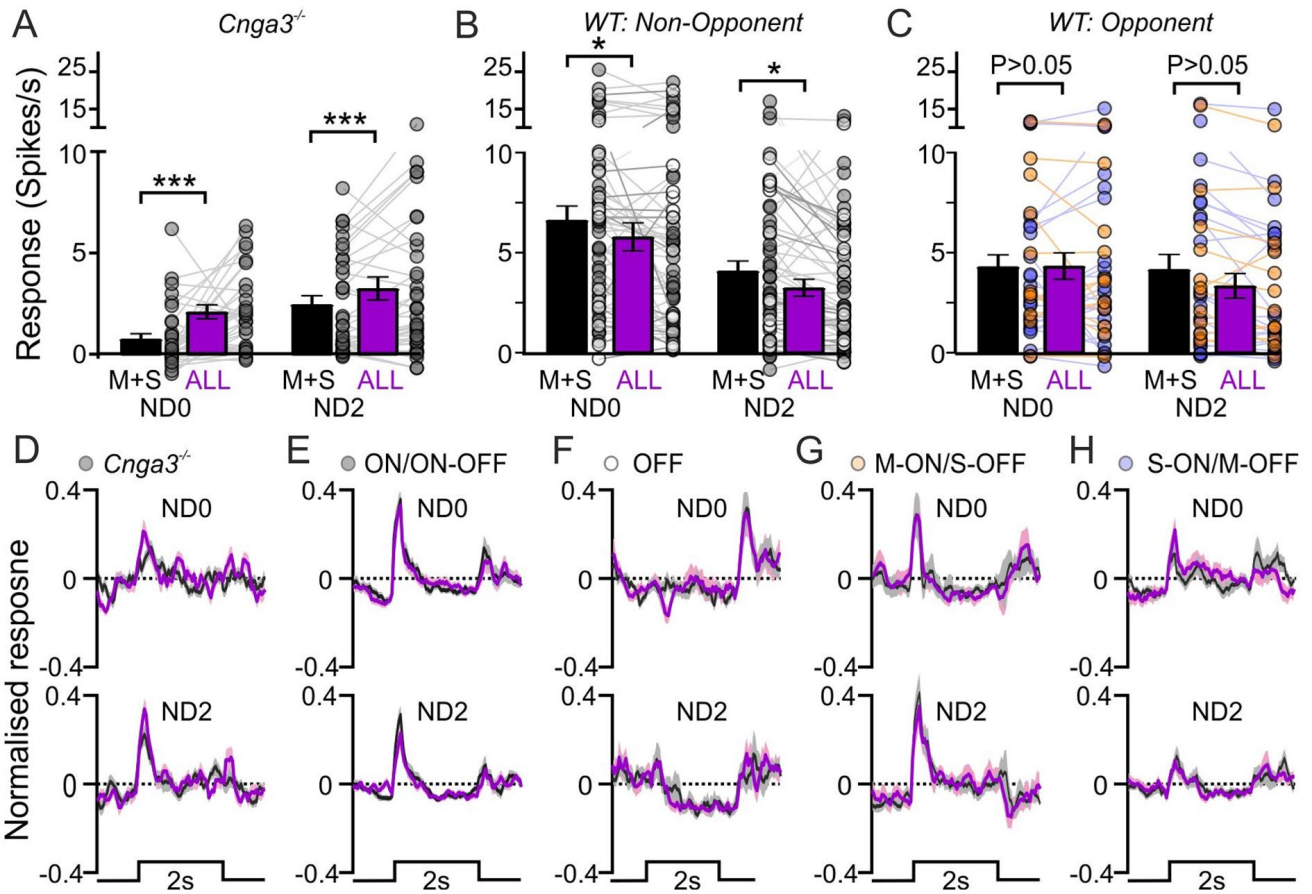
Minimal intrusion in mouse LGN neuron responses to cone directed stimuli under photopic conditions. (**A**–**C**) Mean ± SEM population (and individual cell) response amplitudes evoked by 63% contrast steps directed to M + S cones or All opsins at ND0 and ND0 for *Cnga3*^*−/−*^ (A; n = 37), wildtype non-opponent neurons (**B**; n = 58) or wildtype colour opponent cells (**C**; n = 35). Data analysed by 2-way RM ANOVA (**A**: Stimulus-F_1, 36_ = 12.3, P = 0.001, ND-F_1, 36_ = 14.6, P = 0.0005, interaction-F_1, 36_ = 2.9, P = 0.096; **B**: Stimulus-F_1, 57_ = 11.5, P = 0.001, ND-F_1, 57_ = 17.7, P < 0.0001, interaction-F_1, 576_ = 0.0, P = 0.986; **C**: Stimulus-F_1, 34_ = 3.5, P = 0.069, ND-F_1, 34_ = 1.2, P = 0.281, interaction-F_1, 34_ = 3.2, P = 0.083) with Sidak’s post-tests. (**D**–**H**) Mean ± SEM normalised population response profiles evoked by 63% contrast steps directed to M + S cones or All opsins at ND0 and ND0 for *Cnga3*^*−/−*^ (**D**; n = 37) or wildtype LGN cells with non-opponent ON or ON–OFF responses (**E**; n = 43), non-opponent OFF responses (**F**; n = 15), M-ON/S-OFF responses (**G**; n = 12) or S-ON/M-OFF responses (**G**; n = 23). Throughout, * and ***P < 0.05 and P < 0.001 respectively.

To more directly address the extent to which rod-intrusion might influence responses to cone directed stimuli under our experimental conditions, we investigated responses to M + S and All-opsin contrast stimuli from the dataset described above in more detail. These stimulus pairs provide identical contrast for M- and S-cone opsin but the All-opsin stimulus provides a substantially higher rod contrast than M + S (63% vs. 28% respectively). Accordingly LGN cells recorded in *Cnga3*^*−/−*^ mice exhibited significantly larger responses to the former at both ND0 and ND2 (Fig. 4A,D). By contrast, despite cell–cell heterogeneity in relative response magnitudes, we found no evidence of a systematic increase in responses to All-opsin vs. M + S stimuli in LGN neurons recorded in wildtype mice. Indeed, for the population of cells classified as non-opponent (n = 58) we, in fact, saw a slight but significant decrease in response amplitudes for the All-opsin stimuli at both background light levels (Fig. 4B,E,F). In the case of cells classified as colour opponent (where the M + S, achromatic luminance, condition represents a suboptimal stimulus type), there were no significant systematic differences in response to the two stimuli at either background (Fig. 4C,G,H). In sum these data indicate that, while mouse rods may retain some capability to respond to the contrast associated with our M-cone directed stimuli, the impact of any such influence on overall responses under our experimental conditions is modest and does not noticeably impact chromatic sensitivity.

### Colour processing in identified neurons in mouse subcortical visual system

Having validated our approach for mapping cone-driven visual responses in animals with native cone spectral sensitivity, we next asked whether the corresponding functionally identified neuronal subpopulations in the mouse subcortical visual system might also reflect phenotypically distinct cell groups. Hence many of the regions where we found significant populations of colour opponent neurons (IGL/vLGN and PON) contain a heterogeneous mixture of neurochemically-defined cell types whose functions remain poorly understood^34,35,42–45^. At the broadest level, this includes subpopulations of cells that are distinguished on the basis of their expression of the major excitatory or inhibitory neurotransmitters in the brain, glutamate or GABA. Here then, we sought to distinguish between these groups by using optogenetic approaches to positively identify GABAergic neurons. To this end we crossed GAD2-cre mice^46^ with a cre-dependent channelrhodopsin2 reporter line (Ai32^47^), to generate GAD-ChR2 animals, where the optogenetic actuator is specifically directed to neurons expressing the GABA biosynthetic enzyme GAD2 (the dominant GAD isoform in visual centres^42,44^).

**Figure 5:**
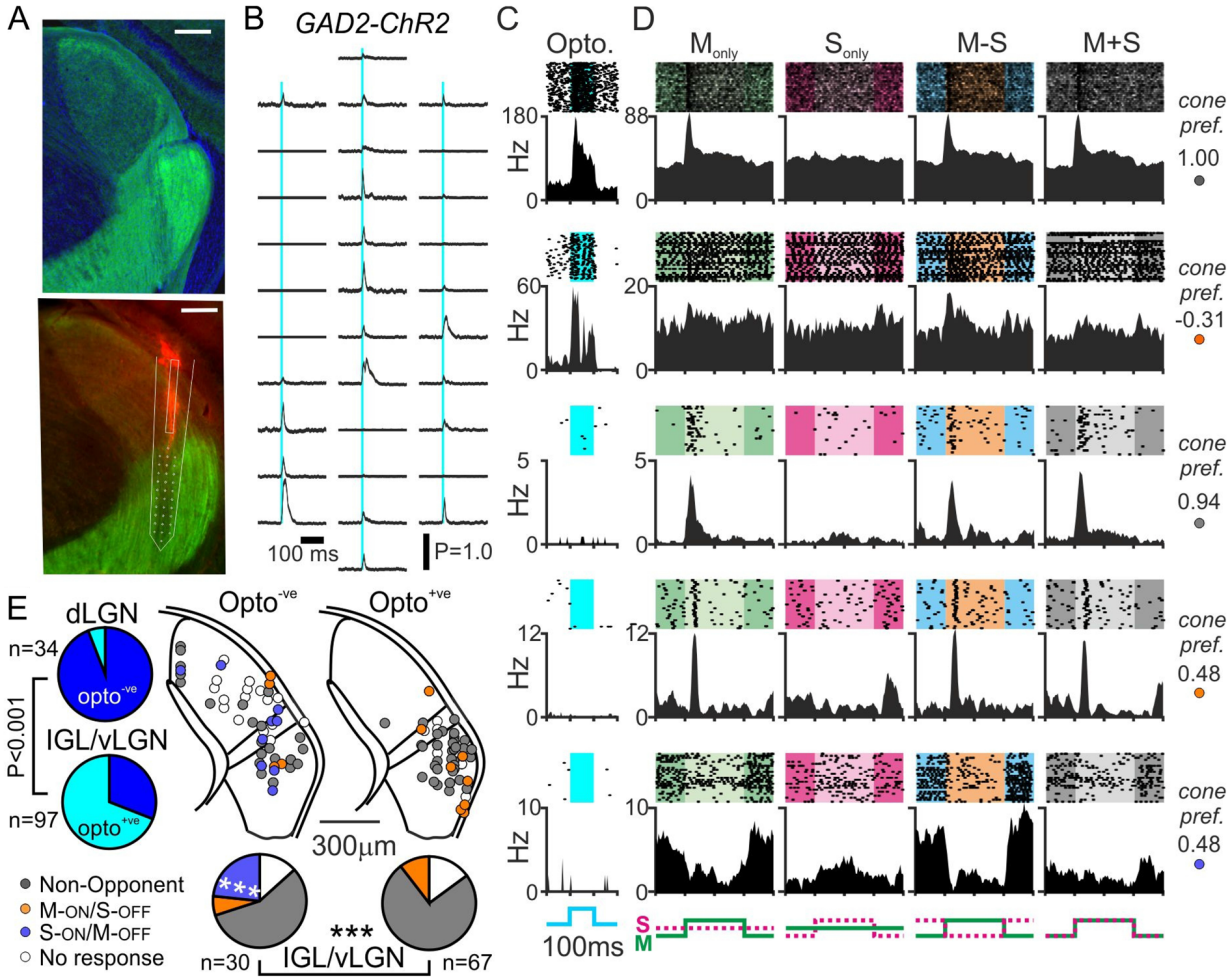
Differential distribution of colour opponent responses between GAD2 positive and negative IGL/vLGN neurons. (**A**) Upper panel shows antibody enhanced ChR2-EYP signal from LGN of GAD2-ChR2 mice (green), co-stained for DAPI (blue). Lower panels shows histology from a GAD2-ChR2 mouse used for optrode recording (ChR2-EYFP: green, Di-I labelled probed track: red). Scale bars = 200 µm. (**B**) Representative multiunit spike responses (mean across 400 trials) to 10 ms, 460 nm, light flash from the 32-channel optrode (corresponding to histology in (**A**): lower). (**C**) Peristimulus histograms and corresponding spike rasters for 100 ms, 460 nm, optogenetic stimulation for five representative IGL/vLGN neurons from GAD2-ChR2 mice (top two classified as optogenetically responsive/GAD2-expressing). (**D**) Responses to 63% contrast cone-modulating stimuli (spike rasters and corresponding histograms) from the same five representative IGL/vLGN LGN neurons classified as (top to bottom): non-opponent, M-ON/S-OFF, non-opponent, M-ON/S-OFF, S-ON/M-OFF. Insets to the right of each set of traces provided the calculated cone preference for each cell ([M_R_ − S_R_]/[M_R_ + S_R_]). (**E**) left panels show pie charts of proportions of dLGN and vLGN neurons from GAD2-ChR2 mice responding to optogenetic stimuli (Opto^+ve^); analysed by Fisher’s exact text. Central panels show projected anatomical location of identified Opto^*−*ve^ and Opto^+ve^ cells identified GAD2-ChR2 mice, colour coded according to cone-driven response properties. Lower pie charts show the proportion of Opto^*−*ve^ and Opto^+ve^ IGL/vLGN neurons exhibiting the various cone-driven response types; data analysed by χ^2^-test followed by Fisher’s exact tests for each category. ***P < 0.001.

We first started by evaluating cone-based responses of identified neurons in multielectrode recordings from the visual thalamus of GAD-ChR2 mice. Here, we principally targeted the IGL/vLGN which, unlike the dLGN which is dominated by excitatory relay neurons, are strongly enriched for GABAergic (GAD2-expressing) neurons^34,35,42,45^ (Fig. 5A). Accordingly, during such recordings, optogenetic stimulation via a fibre attached to the recording electrode (see “Methods” for further details) reliably drove rapid and reliable increases in firing activity across the spatial extent of our 32-channel recording arrays (Fig. 5B). We were therefore able to reliably distinguish GABAergic, Opto^+ve^, neurons from non-GABAergic (putative glutamatergic^45,48,49^) cells (Fig. 5C) and assess the visual response properties (Fig. 5D). In total we isolated n = 131 light responsive LGN neurons from such recordings, principally from the IGL/vLGN (n = 97) although we also recorded a smaller group of dLGN cells (n = 34) via subset of recordings where we placed electrodes partly or wholly in the dLGN (n = 4/10 recordings). Consistent with previous estimates of the prevalence of inhibitory dLGN interneurons (~ 6%)^50^, cells isolated from those dLGN recordings only very rarely responded to optogenetic stimulation (n = 2/34; ~ 6%). By contrast, the majority of IGL/vLGN cells were Opto^+ve^ (n = 67/97; ~ 69%), in line with previous estimates of the proportion of GAD2-expressing cells in retinorecipient portions of the IGL/vLGN^42,48^. The majority of IGL/vLGN cells detected in these experiments responded to our cone-directed stimuli (n = 83/97; ~ 86%) and, interestingly, while both Opto^+ve^ and Opto^−ve^ groups contained examples of colour opponent and non-opponent neurons there was a pronounced difference in the distribution of response types (Fig. 5D,E; χ^2^-test, P < 0.001). Specifically, we found that a high proportion of Opto^*−*ve^ cells (n = 7/30; ~ 23%) exhibited S-ON/M-OFF colour opponency, whereas none of the 67 Opto^+ve^ cells displayed this property (Fishers-exact test, P = 0.0006). Hence, S-ON/M-OFF colour opponency is primarily (and perhaps exclusively), a property of non-GABAergic cells in IGL/vLGN.

**Figure 6:**
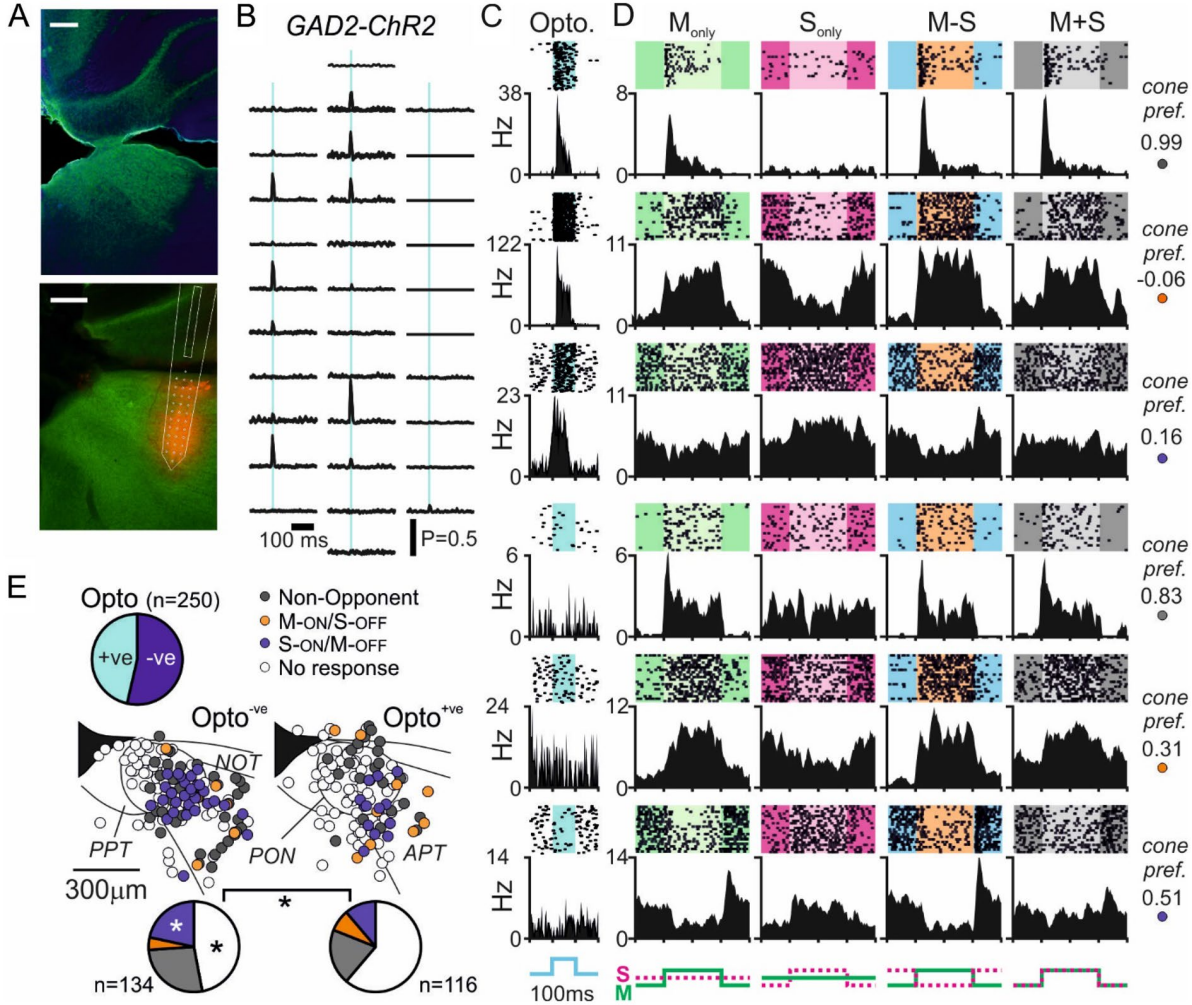
Differential distribution of colour opponent responses between GAD2 positive and negative pretectal neurons. (**A**) Upper panel shows antibody enhanced ChR2-EYP signal from pretectum of GAD2-ChR2 mice (green), co-stained for DAPI (blue). Lower panels shows histology from a GAD2-ChR2 mouse used for optrode recording (ChR2-EYFP: green, Di-I labelled probed track: red). Scale bars = 200 µm. (**B**) Representative multiunit spike responses (mean across 400 trials) to 10 ms, 460 nm, light flash from the 32-channel optrode (corresponding to histology in (**A**): lower). (**C**) Peristimulus histograms and corresponding spike rasters for 100 ms, 460 nm, optogenetic stimulation for six representative pretectal neurons from GAD2-ChR2 mice (top three classified as optogenetically responsive/GAD2-expressing). (**D**) Responses to 63% contrast cone-modulating stimuli (spike rasters and corresponding histograms) from the same six representative IGL/vLGN LGN neurons classified as (top to bottom): non-opponent, M-ON/S-OFF, S-ON/M-OFF, non-opponent, M-ON/S-OFF, S-ON/M-OFF. Insets to the right of each set of traces provided the calculated cone preference for each cell ([M_R_ − S_R_]/[M_R_ + S_R_]). (**E**) Top: pie chart of proportion of pretectal neurons from GAD2-ChR2 mice responding to optogenetic stimuli (Opto^+ve^). Central panels show projected anatomical location of identified Opto^*−*ve^ and Opto^+ve^ cells identified GAD2-ChR2 mice, colour coded according to cone-driven response properties. Lower pie charts show the proportion of Opto^*−*ve^ and Opto^+ve^ pretectal neurons exhibiting the various cone-driven response types; data analysed by χ^2^-test followed by Fisher’s exact tests for each category. *P < 0.001.

Given these findings, we next asked whether there were also differences in the cone-specific responses of GABAergic and non-GABAergic cells in the pretectum where, as for IGL/vLGN, retinorecipient regions contain substantial numbers of GAD2-expressing cells^43–45^ (Fig. 6A). Accordingly, in multielectrode recordings from the PON and surrounding pretectal regions we observed rapid excitatory responses to optogenetic stimulation at subsets of recording sites distributed across our recording array (Fig. 6B) and mapped the cone specific responses of isolated Opto^+ve^ and Opto^*−*ve^ neurons (Fig. 6C,D). In total, we isolated 250 light responsive pretectal neurons (from 11 recordings in GAD-ChR2 mice) of which almost half (n = 116; ~ 46%) were Opto^+ve^. Here again, while we found examples of non-opponent and colour opponent neurons in both groups of pretectal cells, there were significant differences in the proportions of cells exhibiting the various classes of responses (Fig. 6D,E; χ^2^-test, P = 0.03). Specifically, we found that optogenetically identified GAD2-expressing cells contained a significantly higher proportion of cells that lacked robust responses to our cone-directed stimuli and a substantially lower proportion of cells that displayed S-ON/M-OFF opponent responses (Fig. 6E; Fisher’s exact tests, both P = 0.03). In keeping with our findings in the IGL/vLGN, S-ON/M-OFF colour opponency seems to be strongly enriched among non-GABAergic, putative excitatory, neurons in the PON and surrounding pretectum.

## Discussion

Collectively our findings provide important new advances that address current gaps in understanding as to how colour signals are processed in the mammalian visual system. By undertaking a large-scale survey of cone-driven responses across two major retinorecipient complexes in the mouse brain, the pretectum and LGN, we show cone opponent processing is remarkably widespread both in key image forming centres (the dLGN) as well as regions with key roles in non-image forming responses (PON, IGL/VLGN). These findings confirm previous suggestions, based on experiments performed in mice with altered cone spectral sensitivity^29,30^ and rule out the possibility that the observations of many cone opponent cells in *Opn1mw*^*R*^ animals reflects alterations in retinal or brain circuit organisation as a consequence of the transgenic manipulation. Until now it has proven challenging to directly test this question in wildtype mice. We now provide an approach that allows for reliable assessment of cone-based responses in mice with native cone function, opening up possibilities for dissecting the functions and roles of brain and retinal circuits processing colour information using the powerful intersectional genetic tools available in this species. Accordingly, here we use optogenetic based cell-identification to provide the first evidence of functional specialisation among visually responsive neurons in non-image forming centres of the mouse brain (the PON and IGL/vLGN), with S-ON/M-OFF type colour opponent responses being specifically enriched among non-GABAergic cells in these regions.

Previous studies of colour processing in the mouse visual system have used approaches that may not reliably isolate cone-specific responses and have produced highly divergent estimates of the prevalence of spectral opponency^13–19^. The majority of such studies have focused on the retina and have suggested between 2% (based on in vivo recording of optic nerve fibres^13^) to 30% (based on targeted calcium imaging^19^) of RGCs show UV-green opponency. To our knowledge, only one previous study has investigated spectral opponency in any of the regions of wildtype mouse brain we recorded from^18^. In that work, ~ 10% of dLGN neurons were found to exhibit UV-green opponency, with green-ON responses especially rare (~ 1% of cells). By contrast, we find ~ 27% of dLGN neurons exhibit cone-dependent spectral opponency (including ~ 10% of cells with M-ON/S-OFF responses), estimates that closely align with those we obtained using similar approaches in *Opn1mw*^*R*^ mice^30^. The substantially lower proportion of opponent dLGN cells found in the earlier study^18^ may, at least, partly derive from the fact that the stimuli used there were not fully cone-isolating, although the lower overall irradiance and/or extent of LGN sampled may also contribute to difference from the present work.

Across a number of the past studies investigating colour opponency in the mouse retina, a variety of potential circuit mechanisms have been proposed^15–17,19^. A common feature of those proposed mechanisms, however, is that opponency arises via a centre-surround mechanism such that, often, the relevant neurons only exhibit detectable colour opponency for widefield stimuli that cover the receptive field (RF) centre and surround. In the present study we used exclusively full field stimuli so we cannot directly assess the extent to which such properties are true also for the colour opponent pretectal and thalamic neurons recorded here. Nonetheless, an earlier study (noted above) did provide evidence consistent with equivalent centre-surround mechanisms providing an origin for colour-opponency in some dLGN neurons^18^. We also previously mapped cone-subtype specific RFs for a subset of LGN neurons in *Opn1mw*^*R*^ mice and found evidence consistent with centre-surround based opponency in many cells^30^. We further, however, found an equivalent proportion of cells whose responses were more consistent with the presence of opponent RF centre and which displayed robust chromatic responses even for small spot stimuli. Having now validated the use of cone-directed stimuli in central recordings in mice with native M-cone opsin, an exciting prospect for the future will be to extend these approaches to spatially patterned stimuli and determine whether the same divergence in RF properties extends also to LGN and pretectal neurons in wildtype mice.

Another key open question in the field has been the extent to which rod-cone opponency might be important for mouse colour vision, with several studies suggesting this as the origin of UV-green spectral opponency at the level of the retina^17,19^. The overlapping spectral sensitives of rod and M-cones has made this hard to directly assess. Indeed, even using multispectral stimuli, it is impossible to generate very high contrast for one photopigment without also providing some contrast for the other. The conventional approach to address this issue, which we employ here, is to work at background light levels where rods should be saturated. Nonetheless, in line with previous reports that rods can continue to function even under very high background light levels^21^, our recordings in *Cnga3*^*−/−*^ animals confirm that weak rod-based responses are likely to persist in some cells under photopic conditions. Importantly, however, we found no evidence that any such rod intrusion made a meaningful difference to our assessment of colour opponency. Hence, (1) the magnitude of rod-based responses to M-cone directed stimuli revealed in *Cnga3*^*−/−*^ animals were very small compared to responses to the same stimuli in wildtype mice, (2) there was no systematic difference in responses of opponent neurons to stimuli providing the same cone contrast but very different rod contrast and (3) the prevalence and properties of LGN and pretectal opponent neurons found here were virtually identical those reported for cells in *Opn1mw*^*R*^ mice recorded under similar conditions but with cone directed stimuli that lacked any rod contrast^29,30^.

In sum, while the present findings can be confidently be ascribed to cone-based opponency, they do not rule out the possibility that rod-cone opponency might be important under other conditions. Indeed here we find some evidence for a modest antagonist impact of rod signals across the population of cells classed as non-opponent. Although any such action is insufficient to provide apparent opponency for the cone-directed stimuli used here, the relative contribution of rods likely changes as a function of background and adaptation state so could become more important under different conditions^21^.

It is also worth noting here that, while our findings regarding the prevalence and properties of colour opponent neurons in the pretectum and LGN closely match those reported previously in *Opn1mw*^*R*^ mice, we do observe some apparent differences across the population of non-opponent cells. Specifically, we find a more evenly balanced population of cells that prefer M- vs. S-cone directed stimuli compared to non-opponent cells in *Opn1mw*^*R*^ mice (where we observed more very strongly S-opsin biased cells). This most likely reflects the fact that, under our experimental conditions, our M-opsin directed stimuli are associated with some rod intrusion in at least some cells, whereas the equivalent L-opsin directed stimuli in in *Opn1mw*^*R*^ mice lack rod contrast. Difference in cone preference could also reflect sampling differences, since the relative impact of M-/L- vs. S-cone opsin signals will vary depending on what retinal location the cells receive input from. In principle, such differences could also arise if there were a modest reduction in L-opsin expression or function in Opn1wm^R^ animals, compared to the native M-cone opsin, although previous studies have not detected any gross neuroanatomical or functional abnormalities of this nature^28,30^. In either case, while our data do not allow us to definitively distinguish between these various possibilities they certainly do not support the possibility of any ‘gain-of-function’ that could explain previous findings around the prevalence or roles of cone opponency in *Opn1mw*^*R*^mice.

With respect to the general questions that remain around the origins of mouse colour vision, silent substitution approaches of the type used here should provide a useful route for future studies to dissect under what circumstances rods might contribute to colour opponency at the electrophysiological level and colour discrimination at the whole animal level, alongside the more traditionally used transgenic approaches. While a clear expectation based on the present data and past related work is that animals lacking cone function (e.g. the *Cnga3*^*−/−*^) should entirely lack any capacity for colour discrimination, an especially informative comparison here could be investigation of animals that selectively lack rod function. Unfortunately, many of the transgenic/mutant lines with selective lesions of rod function experience widespread cone-degeneration that limits their utility for this purpose (e.g. *Rho*^*−/−*^ or *rd/rd* ^51,52^). Conversely, while this issue is avoided in mice lacking rod-specific transducin (*Gnat1*^*−/−*^), such animals in fact retain some rod-based responses due to a compensatory actions of Gnat2^53^. Hence, while differences in colour discrimination capabilities between *Gnat1*^*−/−*^ and appropriate wildtype controls could, in principle, confirm conditions under which rods contribute to opponency, the retention of colour discrimination in such animals would less reliably rule out rod contributions.

In addition to validating new tools for investigating photoreceptor contributions to mouse vision, the present findings also provide new insight relevant to the almost entirely unexplored questions around the identities and potential functions of neurons in the mouse brain that process colour signals. This question is especially pertinent with respect to non-image forming centres such as the PON and IGL/vLGN which connect widely to many brain regions and possess a mixture of neurochemically defined cell types^34–36,42,45^.

The IGL/vLGN contains an especially diverse compliment of cell types, most of which are GABAergic although some glutamatergic neurons are also present^34,42,45^. Among the many known projection targets of the IGL/vLGN, GABAergic projections have been neuroanatomically and/or functionally identified for several regions including the suprachiasmatic nucleus, superior colliculus, lateral habenula and nucleus reuniens (NRe)^34,35,48,49,54–56^. Our finding that a subset of IGL/vLGN GAD2-expressing cells exhibit M-ON/S-OFF opponency therefore raise the possibility that colour signals provided by such cells could influence aspects of the known functions ascribed to these nuclei, including regulation of the circadian function, reward/mood related processing, memory and executive function or visually guided behavioural reflexes. More strikingly however, we found that none of the optogenetically identified GABA cells exhibited the opposite, S-ON/M-OFF, colour opponent responses, while that property was common among Opto^*−*ve^ cells.

At present there is comparatively little understanding of the connectivity of non-GABAergic cells in the IGL/vLGN region. We previously reported a subpopulation of cells in the IGL/vLGN region that exhibited excitatory responses to electrical stimulation of the contralateral IGL/vLGN^57^, suggesting that some glutamatergic cells are involved in commissural communication between these nuclei (potentially relating to vestibular functions ascribed to this region^34^). More recently, studies have also provided evidence consistent with substantial glutamatergic input to the NRe via the IGL/vLGN. Accordingly, many NRe-projecting IGL/vLGN neurons do not express GAD2, especially around the medial edge of the vLGN external segment where we find many S-ON/M-OFF cells^48^. Moreover, optogenetic stimulation of IGL/vLGN terminals in the NRe evokes primarily glutamatergic excitatory postsynaptic currents, indicating a substantial component of innervation derives from glutamatergic cells^49^. Of course, we cannot definitively ascertain whether the Opto^*−*ve^ cells we recorded in GAD2-ChR2 mice are indeed glutamatergic neurons. While the GAD2-line reliably labels GAD2-expressing cells^46^ and GAD2 is the dominant isoform in visual regions (including LGN and pretectum^42,44^) it remains possible that some GABAergic neurons might lack GAD2 and instead express GAD1. In either case, the results described above raise the interesting possibility that the S-ON/M-OFF colour signals we find in non-GAD2-expressing cells are especially significant for regulation of one or more function ascribed to the NRe which included regulation of memory, executive function and defensive behavioural reflexes^48,49,58^.

In the case of our pretectal recordings, a region which contains more of a mixture of glutamatergic and GABAergic cells^43–45^, we again found S-ON/M-OFF colour opponency to be especially commonplace among non-GAD2-expressing cells, although here we also find evidence for some such responses among GABAergic cells. We especially focused our recordings here on the PON which is known to play a critical role in regulation of the pupil light reflex, via excitatory projections to the Edinger Westphal nucleus^36^. While our data here establish many Opto^*−*ve^ (presumably glutamatergic) cells display S-ON/M-OFF colour opponency, we think it unlikely these are involved in pupil control. Hence, while blue-yellow colour signals are known to influence pupil responses in humans^59–61^, our previous work did not reveal any colour opponency at the level of pupil responses in *Opn1mw*^*R*^ mice^29^. Hence, colour responses identified here among Opto^*−*ve^ cells likely reflect cells involved in other functions ascribed to the PON and surrounding pretectal regions. In this regard it is noteworthy that, while projections from M1 ipRGCs to the PON shell are critical for pupillary responses^62^, the PON core receives input from a variety of different RGC types and seems to play a quite distinct (albeit largely unknown) role in visual function^63^. We further note that we find a fair overall proportion of Opto^+ve^ cells that display colour opponency of one form or other. In addition to local interneurons, the PON also seems to contain long-range projecting GABAergic cells^43,64^. Collectively then, our data suggest significant potential for colour signals relayed via excitatory or inhibitory pretectal cells to influence putative functions such as circadian system and sleep regulation, light evoked blink and other oculomotor functions.

In conclusion our data provide new insight into colour processing in the mouse early visual system, demonstrating the widespread appearance of cone-dependent colour opponent responses across key nuclei of the image-forming and non-image forming visual systems and providing the first evidence for functional specialisation of colour processing to specific cell types. Moreover, the paradigms established and validated here provide a useful tool to underpin future studies seeking to define the organisation, function and roles of neural circuits supporting effects of colour on mouse physiology and behaviour.

## Materials and methods

### Animals

All experiments were performed in accordance with the Animals (Scientific Procedures) Act of 1986 (United Kingdom), received University of Manchester Animal Welfare Ethical Review Body and UK Home Office approval and are reported in compliance with ARRIVE guidelines. Mice were bred and housed at the University of Manchester in a 12:12 h light dark cycle at 22 °C with food and water available ad libitum. Experiments were performed in adult (60–180 days old) male wildtype (C57BL/6J background), *Cnga3*^*−/−*^^40^ or GAD-ChR2 mice^54^. The latter were generated by crossing GAD2-IRES-Cre mice (Jackson Laboratories, strain#: 010802)^46^ with the Ai32 reporter line (Jackson Laboratories, strain#: 012569)^47^ to produce GAD2^cre/+^; Ai32^+/*−*^ experimental animals.

### In vivo electrophysiology

Mice were anaesthetised with urethane (1.55 g/kg i.p; Sigma-Aldrich, Dorset, UK) and prepared for stereotaxic surgery as described previously^29,30^. In brief, a craniotomy (< 1 mm diameter) was placed above the target region (1.1 mm lateral and 2.7 mm posterior to the bregma for pretectal recordings, 2.3 mm lateral and 2.5 mm posterior to bregma for LGN recordings). Atropine (1% in saline; Sigma-Aldrich) was applied to the eyes to dilate the pupils and a drop of mineral oil (Sigma-Aldrich) applied subsequently to retain corneal moisture. Recordings employed 15 µm thick, 32-site, silicon-substrate multielectrode arrays (NeuroNexus, MI, USA) with either 4 shanks each with 8 sites (A4 × 2-tet-150–200-121 or A4 × 8-5 mm-200–50-177) or a single shank polytrode configuration (A1 × 32-5 mm-Poly3-50–177). For studies using optogenetic stimulation the polytrode had a 62.5 µm diameter, 0.22 NA, etched fibre attached 100 µm above the dorsal-most site. In all cases, immediately prior to insertion, recording probes were coated in CM-DiI (V22888; Fisher Scientific, Loughborough, UK) to facilitate post-hoc visualisation in histological images, before being inserted into the brain to target the PON or LGN. Prior to neurophysiological recording, mice were left for at least 30 min to dark adapt and allow neural activity to stabilise.

During recordings, wideband neuronal data was acquired using a Recorder64 data system (Plexon, TX, USA), amplified (3500X), digitised at 40 kHz and stored continuously in a 16bit format. Single unit activity was isolated offline using an automated template-matching based algorithm (Kilosort^65^) and identified clusters and unassigned multiunit spikes were then exported to Offline Sorter (Plexon), as ‘virtual tetrodes’ (spike waveforms detected across 4 adjacent channels) for manual refinement^29^. Single unit isolation was confirmed by reference to MANOVA F statistics, J3 and Davies-Bouldin validity metrics and the presence of a distinct refractory period (> 1.5 ms) in the interspike interval distribution.

### Visual and optogenetic stimulation

Light measurements were performed using a calibrated spectroradiometer (Bentham instruments, Reading, UK). Subsequent quantification as effective photon flux for each class of mouse retinal opsin was then performed by reference to the known opsin sensitivities after correction for prereceptoral filtering^6,22^ as described previously^66^.

Stimuli were generated via a custom source (components from Thorlabs: NJ, USA and Edmund Optics; York, UK) which combined light from three LEDs (λ_max_ 405 nm, 460 nm and 620 nm) via dichroic mirrors. The polychromatic output was delivered via a 7 mm diameter flexible fibre optic light guide positioned 5 mm from the mouse’s contralateral eye and enclosed within an internally reflective plastic cone to provide approximately full field illumination. An equivalent assembly was positioned over the ipsilateral eye, providing light (where required) from a single 405 nm LED. LED intensity was controlled dynamically via a PC running LabVIEW and a USB-6343 DAQ board (National Instruments, TX, USA) and, where required via neutral density (ND) filter wheels to provide a spectrally neutral, 100-fold decrease in light intensity (ND2).

Prior to delivering the visual stimuli described in the manuscript, we evaluated responses to a sequence of 5 s, 405 nm, light steps applied from a background of darkness to the contralateral eye (10 repeats each at intensities of ~ 10^13.8^–10^15.8^ effective photons/cm^2^/s). Cells responding to this stimulus, which should robustly activate visually responsive neurons regardless of which photoreceptor classes they receive input from^54,57,66,67^, were considered light responsive for further analyses. For generation of cone-directed stimuli, we calibrated the three-primary system to re-create ‘white’ light as experienced by mice (i.e. the pattern of photoreceptor activation produced by natural daylight; effective irradiance was 13.8, 14.6, 14.3 and 14.2 log effective photons for S-opsin, M-opsin, melanopsin and rhodopsin respectively). We then adjusted the spectra (via independently modulating brightness of each LED) so as to change activation of M- and/or S- cone opsins, in isolation, unison or antiphase, by ± 63% relative to the background (equivalent to a 0.64 log unit or 4.4-fold change in apparent brightness for the stimulated opsins; Fig. S1B). Stimuli were designed to keep contrast for the silenced cone (where relevant) < 0.3% and to keep melanopsin contrast < 3.5%. M-cone stimuli were associated with nominal modest rod contrasts, however (28–35% Michelson; Fig. S1B). Stimuli were applied as 0.25 Hz square-wave modulations (with a smooth 40 ms transition between ‘bright’ and ‘dim’ phases) and presented as interleaved blocks of 6 cycles of each stimulus (including also spectrally neutral modulations in LED intensity up to 96% contrast). The full protocol was then repeated 5 times to provide 30 repeats for each stimulus. In some experiments we applied the protocol described above initially at 100-fold reduced light intensity (ND2), before presenting under high background light levels.

For optogenetics studies, before and after recording visual responses we assessed responses to trains of blue light flashes (100 flashes each at durations 3–300 ms, interstimulus interval 1 s) produced via a PlexBright 465 nm LED module (Plexon), providing ~ 630 mW/mm^2^ light energy at the fibre tip.

### Histology and immunohistochemistry

After each experiment, brains were removed and placed into 4% paraformaldehyde for 48 h before overnight cryoprotection in 30% sucrose. Brains were then frozen with dry ice and sectioned coronally (width = 100 µm) using a freezing sledge microtome before mounting with Vectashield (Vector laboratories, UK) to glass slides and cover slipping. Sections were imaged under an upright light microscope (BX51; Olympus, UK) with appropriate filter sets for visualisation of DiI fluorescence and images acquired with a Coolsnap HQ camera (Photometrics, USA). Resulting images were scaled and aligned with best matching coronal panels from the mouse atlas^68^ with the anatomical location for each cell estimated based on the known geometry of the recording array and the corresponding recording site location were largest spike amplitudes were detected. For display, estimated unit locations were mapped onto a single anatomical template of the LGN or pretectum. For visualization of ChR2-EYFP expression in GAD2-ChR2 mice, sections were prepared as described above, washed three times in 1% Triton-X100 in PBS (PBS-T) for 5 min each and then incubated in blocking solution (10% normal donkey serum, 0.2% PBS-T) for 1 h at room temperature (21 °C). Sections were subsequently incubated in chicken anti-GFP primary antibody (1:1000, diluted in blocking solution; ab13970; Abcam, Cambridge, MA, USA) overnight at 4 °C. After incubation, sections were washed 3 × 15 min in 0.2% PBS-T and then incubated in Alexa Fluor 488-AffiniPure donkey anti-chicken secondary antibody (dilution 1:5000, diluted in blocking solution; 703–545-155-JIR; Stratech, Singapore, Singapore) for 1 h at room temperature. Sections were washed 3 × 15 min in PBS, mounted onto glass slides with DAPI-containing Vectashield HardSet (H-1500; Vector Laboratories, Inc., Burlingame, CA, USA) and coverslipped. Epifluorescence images of immunostaining were then acquired using a 250 Flash II slide scanner (Pannoramic; 3DHISTECH).

### Data analysis

For analysis of neuronal responses to cone-directed stimuli, spike counts were binned (100 bins/stimulus cycle; smoothed with a 5-bin boxcar filter) and peak-trough amplitudes extracted. To remove the effect of random variations in baseline firing, we derived equivalent peak-trough estimates based on shuffled data (spike counts shuffled in time independently for each trial). Cells were considered responsive based on χ^2^-periodogram analysis (P < 0.05), as described previously^29^, and when the measured response amplitude exceed the 95% confidence limits of responses assessed from shuffled data (100 repeats). The mean shuffled response was subsequently subtracted from the true response such that for non-responsive cells mean amplitude was ≈ 0. Where relevant, response polarity (ON vs. OFF) was assessed based on the stimulus phase where we observed the largest absolute deviation in spike rates from the mean and the sign (positive vs. negative) of that response. Cells were designated as colour opponent where we observed significant responses (as defined above) of opposite sign to S_Only_ and M_Only_ contrast or in cases where responses to one of the two opsins was not detectable but the mean response to the M − S stimuli (analysed above) was significantly greater than that for the M + S stimulus (t-test, P < 0.05). For some analyses, response amplitude (signed by convention as negative for OFF responses) was normalised as a function of variation in the peristimulus time histogram ([max − min]/[min + max]) to give a value between − 1 (maximal OFF) and + 1 (maximal ON). Similarly, for assessment of preference towards M- and S-cone directed stimuli we calculated a standard preference index of the form ([M_Only _− S_Only_]/[M_Only_ + S_Only_]) to give a value between − 1 (Strong S-opsin bias) and + 1 (Strong M-opsin bias).

For comparison of data obtained in wildtype and Opn1mw^R^mice, we used data from our previously published studies^29,38^ collected under closely matched conditions (equivalent irradiance and similar cone contrasts) and analysed using identical procedures to those described above. For analysis of cell densities and response preferences as a function of LGN location, cells were binned based on estimated anatomical location (as described above) using a moving circular window of radius = 150 µm. Within each bin we then calculated the percentage of cells of the relevant type or the average of responses preference, as appropriate. For analysis of responses to optogenetic stimulation, cells were considered Opto^+ve^ where average spike counts during the 3–300 ms stimulation window exceeded the 99% confidence limits of the baseline spike counts (200 ms window preceding stimulus, based on 100–200 trials) for, at least, all stimulus durations > 30 ms.

Higher level statistical comparison were performed using GraphPad Prism v.7 (GraphPad Inc., CA,USA). Most analyses employed one or two way ANOVA, with repeated measures as appropriate, and Sidak’s post-tests where ANOVA revealed main effects of relevant independent variables. Comparisons of proportions of cells employed χ^2^-tests, followed by Fisher’s exact tests for individual response classes as appropriate. Throughout, unless otherwise specified, population data are presented as Mean ± SEM.

## Supplementary Information

Supplementary Figures.

## Data Availability

The datasets generated during and/or analysed during the current study are available from the corresponding author on reasonable request.
