# Supplementary material for: Colour opponency is widespread across the mouse subcortical visual system and differentially targets GABAergic and non-GABAergic neurons
Source: Sci Rep. 2023 Jun 8;13:9313. doi: 10.1038/s41598-023-35885-z (PMC10250360; doi:10.1038/s41598-023-35885-z)
Supplement: Supplementary file 1 — Supplementary Figures. [file 41598_2023_35885_MOESM1_ESM.docx]

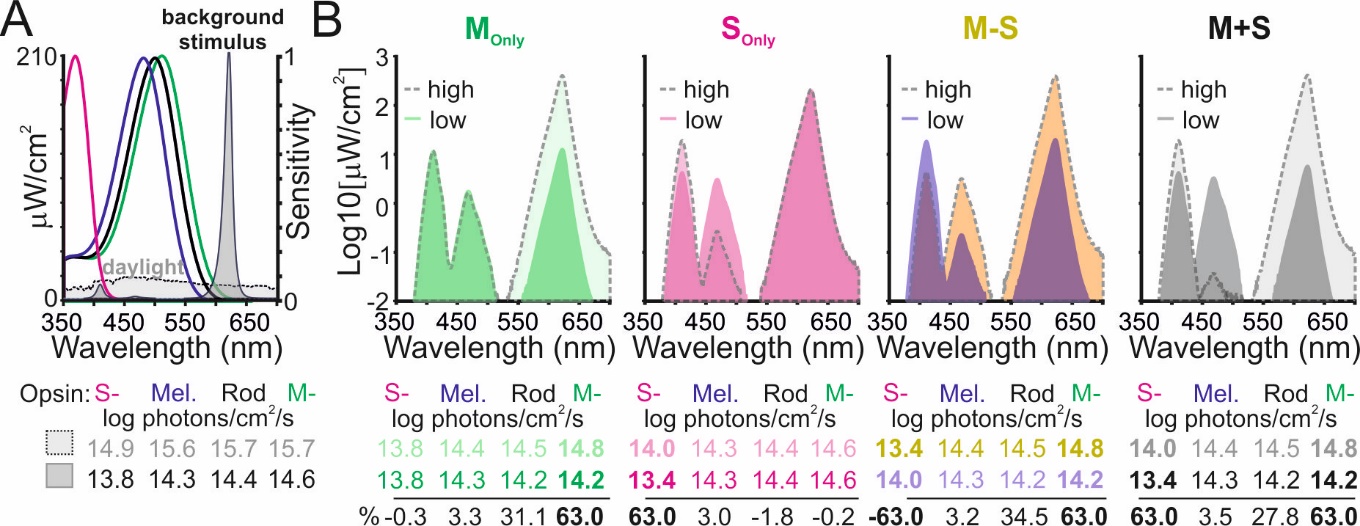


**Supplementary Figure S1. Stimuli used for selective modulation of cone responses and identification of melanopsin responsive neurons.** (**A**) Spectral sensitivity of native mouse opsins (corrected for pre-receptoral filtering) and spectral irradiance of the background stimulus used for neurophysiological recordings, with comparison to a natural (overcast) daylight spectrum quantified according effective photon flux for mouse opsins. (**B**) Spectra (log irradiance scale) and quantification for stimulus pairs designed to provide 63% Michelson contrast for M- and/or S-cone opsin. Transitions between spectra respectively constitute (left to right): ‘M_Only_’, ‘S_Only_’, ‘M-S’ and ‘M+S’ stimuli. Note that, due to their close spectral overlap, M-opsin contrasts are associated with a modest (nominal) rod contrasts, but are presented here at irradiances above the conventional rod saturation point.


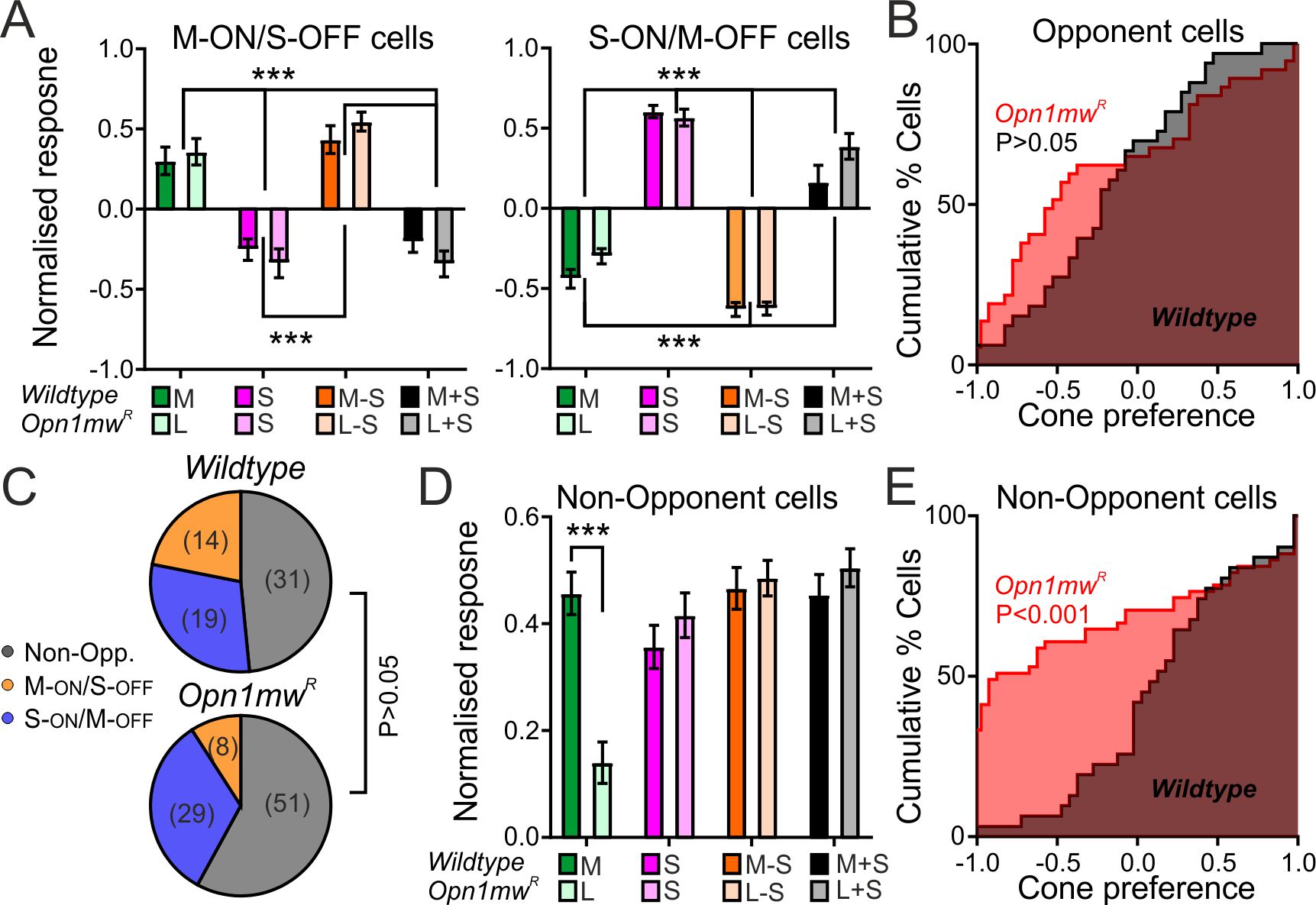


**Supplementary Figure S2. Pretectal responses to cone-directed stimuli in mice with native and altered cone spectral sensitivity.** (**A**) Mean±SEM normalised responses of pretectal cells with M-ON/S-OFF (left) or S-ON/M-OFF type opponency in wildtype and *Opn1mw^R^* mice. Stimuli in wildtype are 63% Michelson contrast (as shown in Fig S1), stimuli in Opn1mw^R^ mice are 60% Michelson contrast, presented on a background of similar irradiance and spectral composition (<0.2 log unit difference in effective photons/cm^2^/s for each opsin class). Data analysed by 2-way mixed-effects ANOVA (**Left:** Stimulus - F_3, 60_ = 40.6, P<0.0001; Genotype - F_1, 20_ = 0.11, P=0.746; Interaction - F_3, 60_ = 0.85, P=0.472; **Right:** Stimulus - F_3, 138_ = 162.2, P<0.0001; Genotype - F_1, 46_ = 3.25, P=0.078; Interaction - F_3, 138_ = 1.87, P=0.138), with Sidak’s post-tests. (**B**) Cumulative frequency plot of cone-opsin preference for wildtype and *Opn1mw^R^* colour opponent neurons (n=33 and n=37 respectively; data for M-ON/S-OFF and S-ON/M-OFF cells combined). Data analysed by Kolmogorov-Smirnov test. (**C**) Proportions of colour opponent and non-opponent cells detected in pretectal recordings from wildtype and *Opn1mw^R^* mice under equivalent conditions. Data analysed by χ^2^-test. (**D**) Mean±SEM normalised responses of non-opponent pretectal cells in wildtype and *Opn1mw^R^* mice for stimuli as described in **B**. Data for cells with non-opponent OFF responses (n=4/31 wildtype and n=10/51 *Opn1mw^R^*, sign inverted). Data analysed by 2-way mixed-effects ANOVA (Stimulus - F_3, 240_ = 13.6, P<0.0001; Genotype - F_1, 80_ = 1.35, P=0.248; Interaction - F_3, 240_ = 15.1, P<0.0001), with Sidak’s post-tests. (**E**) Cumulative frequency plot of cone-opsin preference for wildtype and *Opn1mw^R^* non-opponent pretectal neurons (n=31 and n=51 respectively). Data analysed by Kolmogorov-Smirnov test. Throughout: *** indicates P<0.001.


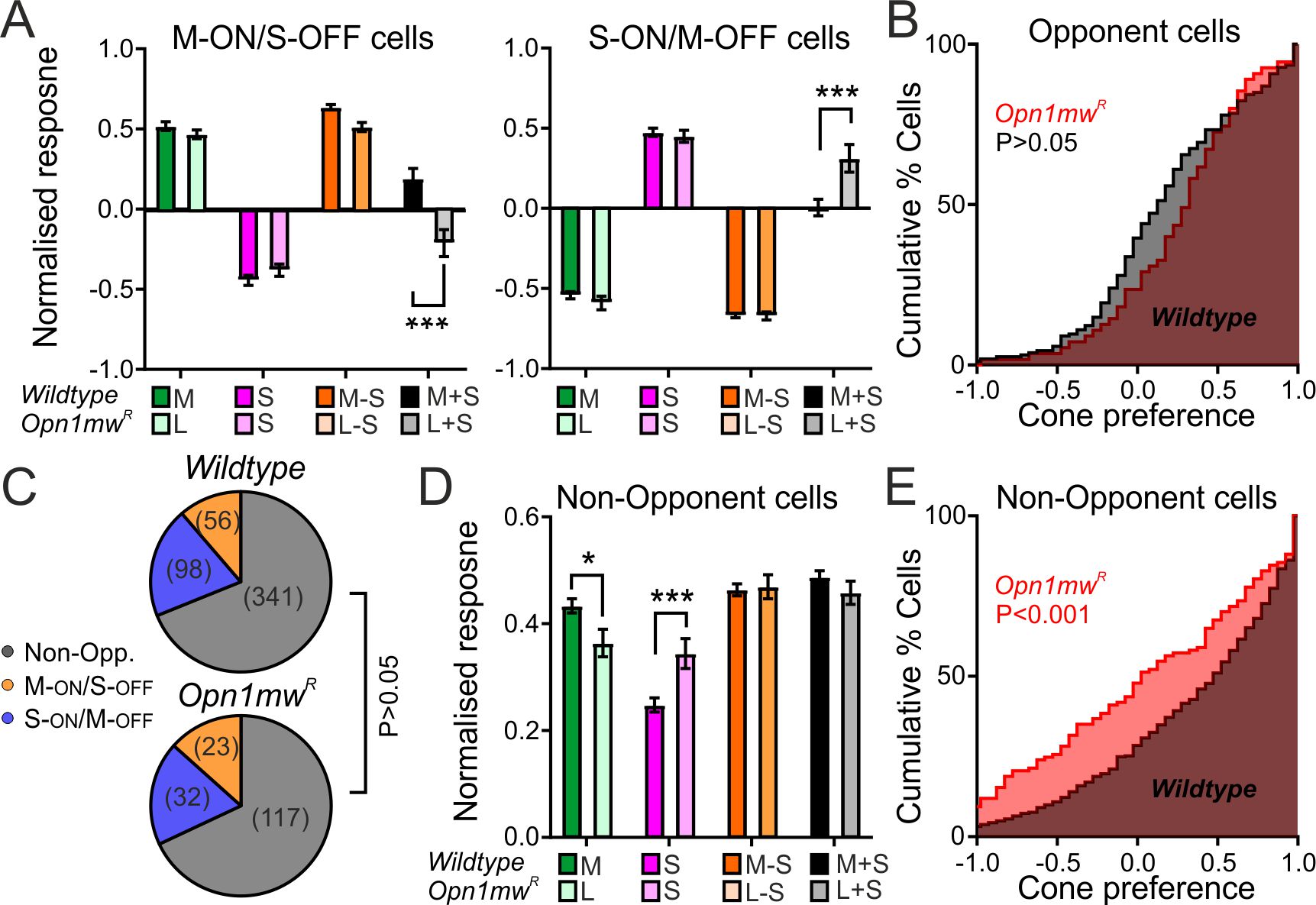


**Supplementary Figure S3. LGN responses to cone-directed stimuli in mice with native and altered cone spectral sensitivity.** (**A**) Mean±SEM normalised responses of LGN cells with M-ON/S-OFF (left) or S-ON/M-OFF type opponency in wildtype and *Opn1mw^R^* mice. Stimuli in wildtype are 63% Michelson contrast (as shown in Fig S1), stimuli in Opn1mw^R^ mice are 75% Michelson contrast, presented on a background of similar irradiance and spectral composition (<0.2 log unit difference in effective photons/cm^2^/s for each opsin class). Data analysed by 2-way mixed-effects ANOVA (**Left:** Stimulus – F_3, 231_ = 188.5, P<0.0001; Genotype - F_1, 77_ = 11.1, P=0.001; Interaction - F_3, 231_ = 9.6, P<0.0001; **Right:** Stimulus - F_3, 384_ = 309, P<0.0001; Genotype - F_1, 128_ = 3.29, P=0.072; Interaction - F_3, 384_ = 7.1, P<0.0001), with Sidak’s post-tests. (**B**) Cumulative frequency plot of cone-opsin preference for wildtype and *Opn1mw^R^* LGN colour opponent neurons (n=154 and n=55 respectively; data for M-ON/S-OFF and S-ON/M-OFF cells combined). Data analysed by Kolmogorov-Smirnov test. (**C**) Proportions of colour opponent and non-opponent cells detected in LGN recordings from wildtype and *Opn1mw^R^* mice under equivalent conditions. Data analysed by χ^2^-test. (**D**) Mean±SEM normalised responses of non-opponent LGN cells in wildtype and *Opn1mw^R^* mice for stimuli as described in **B**. Data for LGN cells with non-opponent OFF responses (n=157/341 wildtype and n=36/117 *Opn1mw^R^*, sign inverted). Data analysed by 2-way mixed-effects ANOVA (Stimulus - F_3, 1368_ = 38.5, P<0.0001; Genotype - F_1, 456_ = 0.003, P=0.953; Interaction - F_3, 1368_ = 6.61, P<0.0001), with Sidak’s post-tests. (**E**) Cumulative frequency plot of cone-opsin preference for wildtype and *Opn1mw^R^* non-opponent LGN neurons (n=341 and n=117 respectively). Data analysed by Kolmogorov-Smirnov test. Throughout: *, *** indicate P<0.05 and P<0.001 respectively.

_
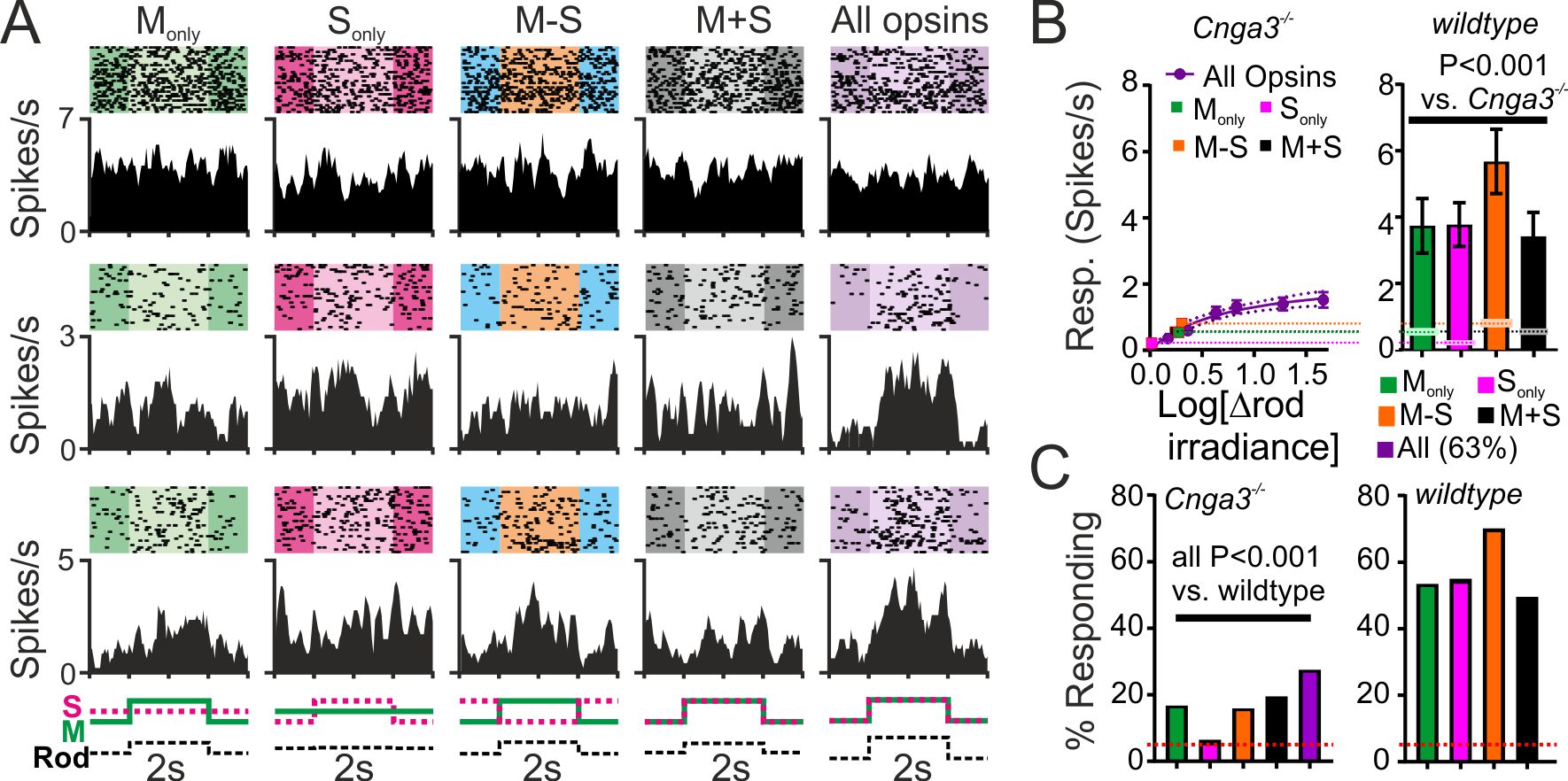
_

**Supplementary Figure S4. Weak rod-driven responses in mouse pretectal neurons under photopic conditions.** (**A**) Responses to full intensity (ND0) 63% contrast cone-modulating and spectrally neutral (‘All opsin’) stimuli from three representative *Cnga3^-/-^* mouse pretectal neurons. Spike rasters and corresponding histograms for each cell plotted on a different horizontal row. (**B**) Left: Mean±SEM responses evoked by cone-modulating stimuli and All opsin contrast (20-96%) plotted as a function of log change in rod-effective irradiance for light responsive pretectal neurons in *Cnga3^-/-^* mice (n=112 cells from 7 recordings). Responses to cone modulating stimuli compared vs. a 2-parameter saturating fit to All opsin contrast response data (extra-sum-of-squares F-test: F_2,1116_=0.34, P=0.71). Right: Mean±SEM responses of wildtype mouse pretectal neurons to cone modulating stimuli (n=78 cells from 14 recordings). Data compared against responses to same stimuli in *Cnga3^-/-^*by two-way mixed effects ANOVA (Genotype: F_1,188_=33.9, P<0.001; Stimulus: F_3,563_=14.2, P<0.001; Interaction: F_3,564_=7.5, P<0.001). (**C**) Proportions of neurons exhibiting significant modulations in firing rate to ND0, 63% contrast, cone-modulating and All opsin stimuli in *Cnga3^-/-^* (left) and wildtype (right) mouse pretectum (same cells as in **B**); proportions responding for each stimulus compared across genotypes by Fisher’s exact test.


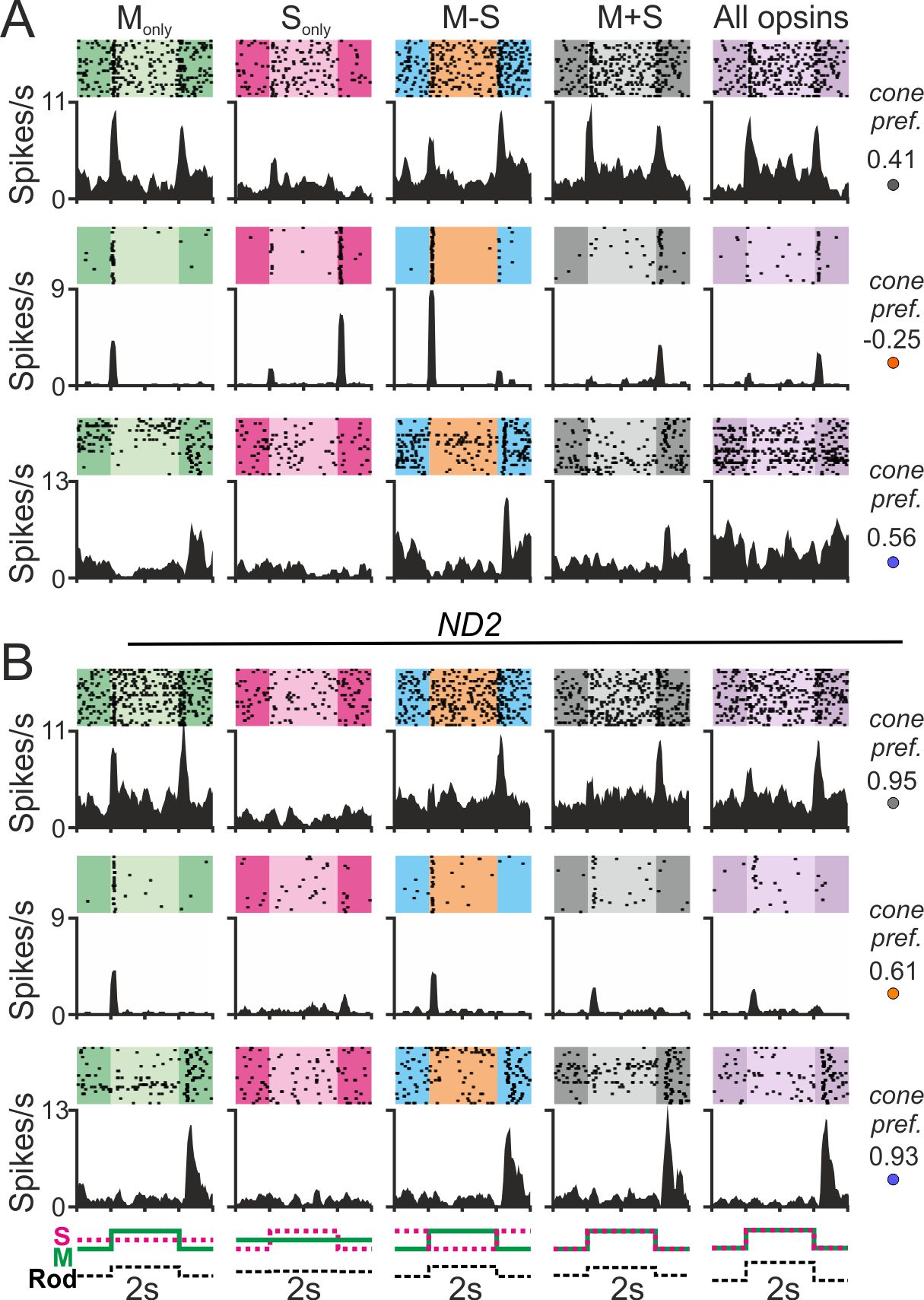


**Supplementary Figure S5. Wildtype mouse LGN neurons responses at high and low photopic intensities.** (**A, B**) Responses to 63% contrast cone-modulating and spectrally neutral (‘All opsin’) stimuli for three representative mouse LGN neurons tested at high photopic (**A**, ND0) and 100-fold dimmer intensity (**B**, ND2) Spike rasters and corresponding histograms for each cell plotted on a different horizontal row; upper panels = non-opponent neuron, middle panels = M-ON/S-OFF opponent neuron; lower panels: S-ON/M-OFF neuron.
